# Nanocarriers for natural polyphenol senotherapeutics

**DOI:** 10.1111/acel.14178

**Published:** 2024-04-29

**Authors:** Natali Joma, Patrick‐Brian Bielawski, Anjali Saini, Ashok Kakkar, Dusica Maysinger

**Affiliations:** ^1^ Department of Pharmacology and Therapeutics McGill University Montreal Quebec Canada; ^2^ Department of Chemistry McGill University Montreal Quebec Canada

**Keywords:** cellular senescence, drug delivery, fisetin, nanocarriers, polyphenols, quercetin, resveratrol, senolytics, senomorphics

## Abstract

Senescence is a heterogenous and dynamic process in which various cell types undergo cell‐cycle arrest due to cellular stressors. While senescence has been implicated in aging and many human pathologies, therapeutic interventions remain inadequate due to the absence of a comprehensive set of biomarkers in a context‐dependent manner. Polyphenols have been investigated as senotherapeutics in both preclinical and clinical settings. However, their use is hindered by limited stability, toxicity, modest bioavailability, and often inadequate concentration at target sites. To address these limitations, nanocarriers such as polymer nanoparticles and lipid vesicles can be utilized to enhance the efficacy of senolytic polyphenols. Focusing on widely studied senolytic agents—specifically fisetin, quercetin, and resveratrol—we provide concise summaries of their physical and chemical properties, along with an overview of preclinical and clinical findings. We also highlight common signaling pathways and potential toxicities associated with these agents. Addressing challenges linked to nanocarriers, we present examples of senotherapeutic delivery to various cell types, both with and without nanocarriers. Finally, continued research and development of senolytic agents and nanocarriers are encouraged to reduce the undesirable effects of senescence on different cell types and organs. This review underscores the need for establishing reliable sets of senescence biomarkers that could assist in evaluating the effectiveness of current and future senotherapeutic candidates and nanocarriers.

## Graphical abstract

1. Cellular senescence is a critical concern in our aging population, and there is a growing need for strategies to modulate senescence.
2. Polyphenols have been explored as senotherapeutics in preclinical and clinical settings. However, their application is hindered by limited stability, modest bioavailability, and insufficient concentrations at target sites.
3. To overcome these limitations, nanocarriers including polymeric nanoparticles and lipid vesicles can be utilized to reduce cytotoxicity, enhance bioavailability, and augment the biological activity of several polyphenols.

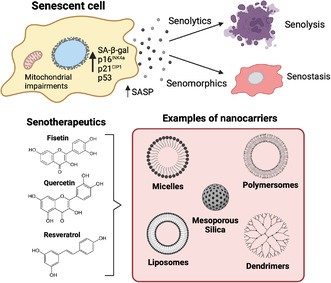

**Abbreviations**
AML: acute myelogenous leukemia
BM‐MSCs: bone marrow‐derived mesenchymal stem cells
FFA: free fatty acids
HUVECs: human umbilical vein endothelial cells
IPF: idiopathic pulmonary fibrosis
IDD: intervertebral disc degeneration
mTOR: mammalian target of rapamycin
MSNs: mesoporous silica nanoparticles
MCI: mild cognitive impairment
MAPK: mitogen‐activated protein kinase
MEFs: murine embryonic fibroblasts
NLCs: nanostructured lipid nanocarriers
ROS: reactive oxygen species
SASP: senescence‐associated secretory phenotype
SCAPs: senescent cell anti‐apoptotic pathways
SOD: superoxide dismutase
WAT: white adipose tissue

## 1 CELLULAR SENESCENCE

### 1.1 Hallmarks of senescence

**FIGURE 1:**
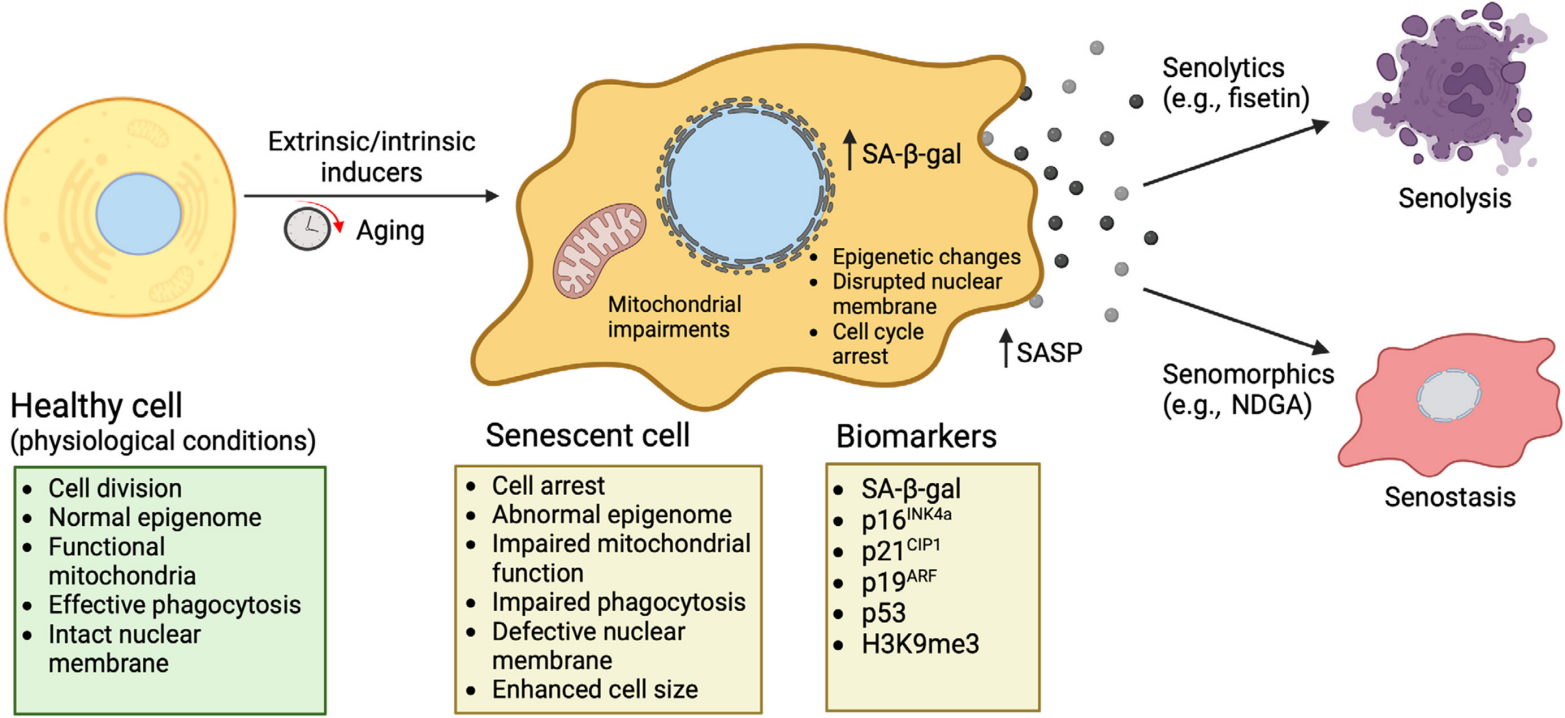
Features of senescence and effects of senolytics and senomorphics. Cellular senescence can be induced through extrinsic/intrinsic inducers or with aging and is characterized by enlarged soma size and irregular cell morphology, an increase in senescence‐associated β‐galactosidase activity (SA‐β‐gal), mitochondrial impairments, epigenetic changes, disrupted nuclear membrane, and the production of the senescence‐associated phenotype (SASP). Two types of senotherapeutics exist: senolytics (e.g., fisetin), which induce senolysis in senescent cells, and senomorphics (e.g., nordihydroguaiaretic acid; NDGA), which reduce the pro‐inflammatory secretory phenotype, causing senostasis. Figure adapted from (Zhang, Pitcher, et al., 2023) and created using BioRender.com.

Cellular senescence is a heterogeneous and dynamic process influenced by genetic, epigenetic, and environmental factors, observed in several types of somatic cells (Reimann et al., 2024). While cellular senescence is involved naturally throughout a human's lifespan in tissue remodeling, embryogenesis, wound repair, and tumor suppression, the accumulation or prolonged presence of senescent cells can lead to age‐related diseases. Senescence is induced by various types of intrinsic and extrinsic stressors, including ionizing and ultraviolet radiation, oncogenic transformation, and mitochondrial dysfunction, contributing to increased reactive oxygen species (ROS) and secretion of pro‐inflammatory mediators—a phenomenon known as senescence‐associated secretory phenotype (SASP) (Figure 1) (Gorgoulis et al., 2019).

The accumulation of senescent cells is associated with multiple pathologies, known as “senopathies,” which encompass cardiovascular diseases, neurodegeneration (Kritsilis et al., 2018), and cancer, among others (Lushchak et al., 2023). These cells exhibit resilience to long‐term DNA damage, oxidative stress, proteotoxicity, and other stressors that induce apoptosis through multiple senescent cell anti‐apoptotic pathways (SCAPs) (Ovadya & Krizhanovsky, 2018). Moreover, senescence plays a dual role in tumorigenesis, acting both as a barrier against tumor development and as a promoter of tumor progression through the secretion of SASP factors (Schmitt et al., 2022). Current preclinical studies predominantly focus on exploring the therapeutic potential of targeting senescence‐related pathways in cancer, with ongoing investigations aiming to understand the efficacy and safety of therapies in modulating the SASP. Phytochemicals, including alkaloids and polyphenols, have demonstrated the ability to promote apoptosis, thereby suppressing tumor growth and cell proliferation (Sa et al., 2023).

Cellular senescence is primarily characterized by permanent growth arrest, with several biochemical and morphological features facilitating identification, including enlarged cell size. As senescence progresses, cells gradually become unresponsive to mitogens and growth factors (Kuilman et al., 2010) but remain metabolically active. They also exhibit increased activity of senescence‐associated β‐galactosidase (SA‐β‐gal), the first marker used for detecting cellular senescence in tissues in situ (Dimri et al., 1995; Kurz et al., 2000). Moreover, senescent cells trigger damage response signaling pathways such as p38 mitogen‐activated protein kinases (p38 MAPK) and nuclear factor kappa‐B (NFκΒ). Senescent cells express $p16^{\mathrm{INK}4A}$ and $p21^{\mathrm{WAF}1/\mathrm{Cip}1}$, leading to the formation of DNA segments with chromatin alterations that reinforce senescence. A brief summary of biomarkers and their limitations is provided in Reimann et al. (2024). It is important to note that there is no singular universal marker for senescent cells, and it may not exist, making it challenging to identify senescence (Gil, 2023). Hence, the development of senolytics primarily focuses on pro‐survival pathways, including B‐cell lymphoma 2 (Bcl‐2), phosphoinositide 3‐kinases (PI3K), p53/p21, tyrosine kinases, hypoxia‐inducible factor‐1α (HIF‐1α), and heat‐shock protein 90 (Hsp90) (Kirkland & Tchkonia, 2017; Ovadya & Krizhanovsky, 2018; Zhu et al., 2015). Several compounds, including those approved by the US Food and Drug Administration (FDA), along with phytochemicals and synthetic compounds, have been proposed as strategies for extending lifespan and mitigating the severity of chronic diseases (Martel et al., 2020; Ngoi et al., 2021).

### 1.2 Senotherapeutics

Senotherapeutics consists of two categories of drugs and natural products: senomorphics and senolytics (Lagoumtzi & Chondrogianni, 2021). Senolytics are compounds designed to selectively eliminate senescent cells, inducing senolysis. The majority of identified senolytics induce apoptosis in senescent cells by targeting critical enzymes involved in pro‐survival and anti‐apoptotic mechanisms, such as p53, p21, FOXO4, PI3K, Bcl‐2 family proteins, Akt, and others. Senomorphics are compounds that suppress/reprogram the SASP or the pro‐inflammatory secretome. Some molecules, however, may be categorized as senomorphics based on their ability to inhibit other biomarkers of cellular senescence without killing senescent cells. Senomorphics work by attenuating the SASPs, leading to senostasis (Figure 1). Generally, senomorphics suppress senescence by targeting key signaling pathways, including NF‐κB, mammalian target of rapamycin (mTOR), IL‐1α, p38 mitogen‐activated protein kinase (MAPK), and others, thereby suppressing SASP expression. Both senolytics and senomorphics have the potential to prevent and treat age‐related disorders, as well as increase life expectancy (Chaib et al., 2022). A summary of some cellular senescence features and the effects of senolytic and senomorphic agents is shown in Figure 1. This review primarily focuses on a selected group of flavonoids and nonflavonoid compounds (dasatinib), tested in vitro, in animal models, and in clinical trials.

Flavonoids are natural polyphenolic compounds commonly found in many fruits and vegetables. Their structure is characterized by a 2‐phenyl‐benzo‐pyrane backbone, consisting of two benzene rings attached to a 3‐carbon unit heterocyclic pyran ring (C6–C3–C6), which is crucial for their classification (Mbara et al., 2022) (Table 1). Flavonoids have been identified as promising candidates for delaying the aging process. Their impact is broad, affecting cellular lifespan, β‐galactosidase activity, and other biomarkers of senescence, both directly and indirectly (Lim et al., 2015; Mbara et al., 2022; Ogrodnik et al., 2017; Yousefzadeh et al., 2018; Zhu et al., 2017). Extensive evidence from experimental animal studies has shown that flavonoids primarily exhibit senomorphic, properties, with only a few displaying senolytic properties (Lagoumtzi & Chondrogianni, 2021; Lim et al., 2015; Xu et al., 2018).

**TABLE 1:** Physicochemical properties of selected senolytic agents and their mechanism of action.

Structure	Compound formula	MW (g⋅mol^−1^)	Aqueous solubility (logP)	A_λmax_/nm	Appearance	Preparation	Senolytic mechanism
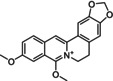	Berberine C_20_H_18_NO_4_	336.4	1.90 mg/mL (3.7)	429	Yellow solid	Natural extract Synthetic	↓ p16
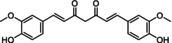	Curcumin C_21_H_20_O_6_	368.4	0.60 μg/mL (3.0)	425	Orange solid	Natural extract Synthetic	↓ NFκB, ↓ mTOR, ↑ SIRT1
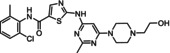	Dasatinib C_22_H_26_CIN_7_O_2_S	488.0	0.008 mg/mL (1.8)	323	White powder	Synthetic	↓ Src tyrosine kinase
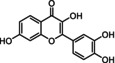	Fisetin C_15_H_10_O_6_	286.2	0.151 mg/mL (1.8)	362	Yellow solid	Natural extract Synthetic	↓ BCL‐2, ↓ PI3K/AKT, ↓ p53, ↓ NFκB
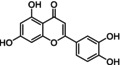	Luteolin C_15_H_10_O_6_	286.2	0.85 mg/mL (3.2)	349	Yellow needles	Natural extract Semi‐synthetic	↓ ROS, ↓ NFκB
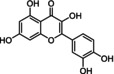	Quercetin C_15_H_10_O_7_	302.2	0.01 mg/mL (1.8)	372	Yellow crystalline solid	Natural extract Semi‐synthetic	↓ ROS, ↓ NFκB, ↓ Bcl‐xL, ↓ PI3K/Akt, ↓ p16, ↓ p21
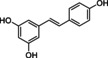	Resveratrol C_14_H_12_O_3_	228.2	0.05 mg/mL (3.1)	*trans*‐321 *cis*‐285	White powder	Natural extract Synthetic	↑ SIRT1, ↓ NFκB, ↑ Nrf2

Flavonoids in general exhibit poor aqueous solubility, stability, short half‐life, and low bioavailability. Their absorption following oral administration and subsequent plasma concentration is very low, with on average, <10% found in urine and plasma (Scalbert & Williamson, 2000). Another challenge in oral administration is the vulnerability of polyphenols in the digestive system, which varies with pH levels and enzyme concentrations. These factors limit the dose effectiveness and therapeutic benefits of polyphenols (Wildman et al., 2006). A variety of nanoformulations have been developed to address these challenges and maximize their therapeutic efficacy. Here, we provide a brief analysis of encapsulating polyphenols into some of these carriers. This review will focus on three senolytic agents: fisetin, quercetin, and resveratrol, but additional senolytics such as berberine, curcumin, luteolin, and dasatinib are also briefly described.

## 2 EXAMPLES OF POLYPHENOLS TESTED IN PRECLINICAL AND CLINICAL STUDIES

### 2.1 Fisetin

**FIGURE 2:**
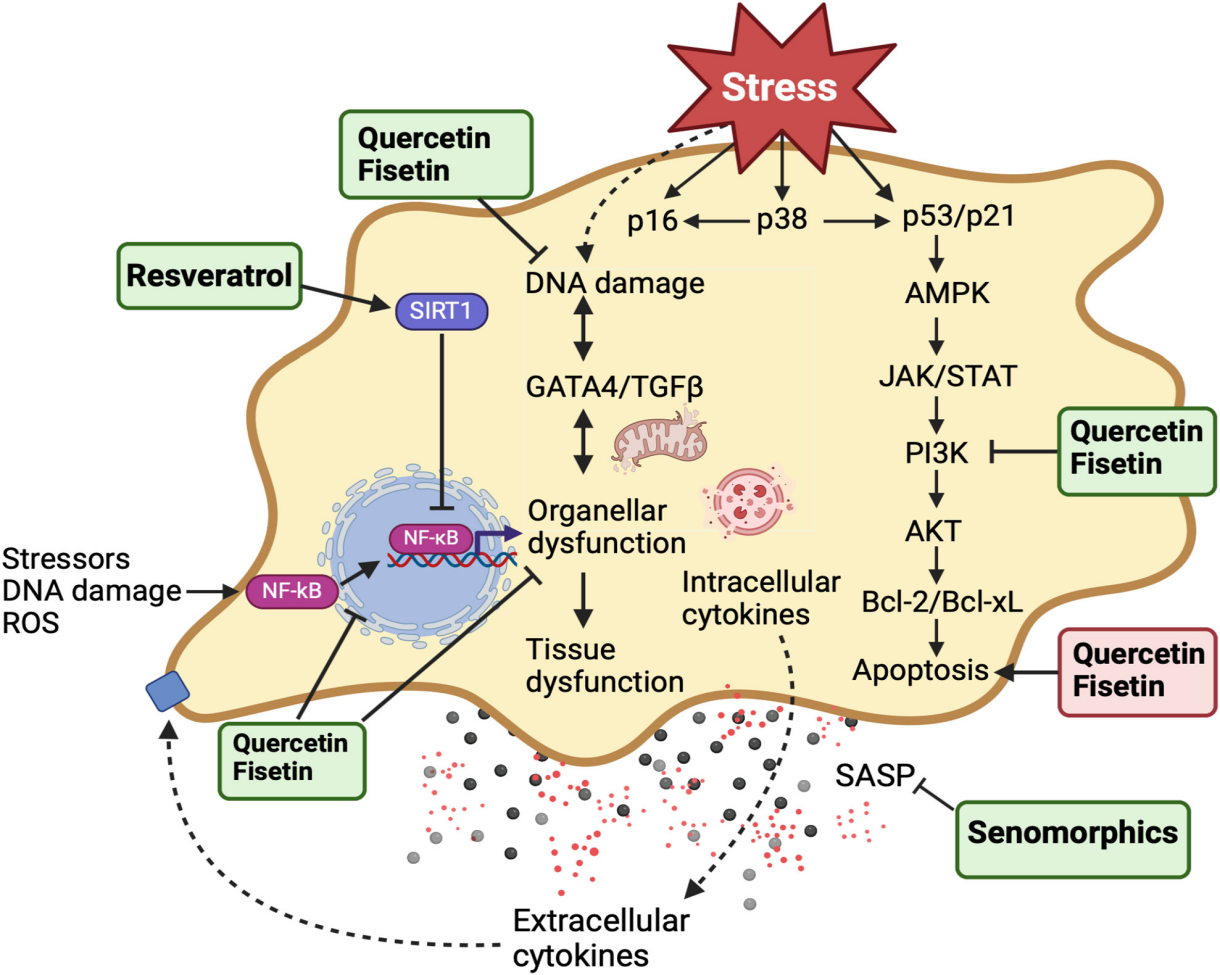
A simplified presentation of the mechanisms of action of fisetin, quercetin, and resveratrol in senescence. Senescent cells, enriched for SASPs, release a varied array of senescence signals (pro‐inflammatory interleukins, chemokines, and damage‐associated molecular patterns) to the neighboring cells. Senolytic agents are shown to suppress markers of senescence and attenuate SASP production. The molecular hallmarks of senescent cells and the pathways regulating SASP production and immune cell infiltration are illustrated. SASP expression is primarily governed by the p38‐MAPK, NF‐κB transcription factors, and BAX/BAK. The green boxes indicate senomorphics, while the red represent senolytics. Created using BioRender.

The flavonol fisetin (7,3′,4′‐flavon‐3‐ol) is a yellow coloring agent found in various plants including onions, strawberries, apples, and persimmons, first isolated from Venetian sumac (Grynkiewicz & Demchuk, 2019). Fisetin plays a role in various biological processes that may contribute to its senolytic effects. Its hydrophobic properties allow it to permeate and accumulate in the cell membrane, leading to antioxidant and anti‐inflammatory effects. Additionally, fisetin induces apoptotic effects in senescent cells by suppressing Bcl‐2 family members and other components of the SCAP network (Figure 2) (Schafer et al., 2017). In wild‐type mice, fisetin reduced senescent cell viability and extended lifespan by inhibiting pro‐senescence effectors such as p16^INK4A^ (Mahoney et al., 2024) and p21^WAF1/Cip1^ (Yousefzadeh et al., 2018). Figure 2 depicts some of the pathways in senescence that are modulated by fisetin.

The therapeutic effects of fisetin are believed to arise from the modulation of NF‐κB and Nrf2 redox‐responsive transcription factors (Sandireddy et al., 2016). Fisetin effectively protected rats with diabetic neuropathy from oxidative damage and neuroinflammation by suppressing NF‐κB and increasing Nrf2 activity (Sandireddy et al., 2016). Moreover, fisetin has been found to eliminate senescent Ercc1^−/−^ murine embryonic fibroblasts (MEFs) and human fibroblasts (IMR90) (Yousefzadeh et al., 2018). Fisetin administration to Ercc1^−/−^ progeroid mice decreased the expression of SASP markers in all tissues and significantly reduced p16‐positive cells in white adipose tissue (WAT) of aged mice (Yousefzadeh et al., 2018). However, p16‐positive macrophages or dendritic cells were unaffected, implying that fisetin's senolytic action is cell type specific. Ex vivo treatment of human adipose explants with fisetin reduced SASP factors (IL‐6, IL‐8, and MCP‐1) and β‐galactosidase positive cells considerably. Multiple ongoing clinical trials are studying the effects of fisetin in various age‐related diseases, including osteoarthritis, frail elderly syndrome, and femoroacetabular impingement, suggesting the potential clinical benefits of fisetin in terms of practicality, safety, and tolerability (Table 2).

**TABLE 2:** Senolytic agents in clinical trials.

Study title	NCT number	Conditions	Interventions
Senolytic agent improves the benefit of platelet‐rich plasma and losartan	NCT05025956	Femoroacetabular impingement	Fisetin
Targeting cellular senescence with senolytics to improve skeletal health in older humans	NCT04313634	Healthy	Dasatinib, quercetin, fisetin
Senolytic drugs attenuate osteoarthritis‐related articular cartilage degeneration: a clinical trial	NCT04210986	Osteoarthritis, Knee	Fisetin
Alleviation by fisetin of frailty, inflammation, and related measures in older adults	NCT03675724	Frail elderly syndrome	Fisetin
Alleviation by fisetin of frailty, inflammation, and related measures in older women	NCT03430037	Frail elderly syndrome	Fisetin
An open‐label intervention trial to reduce senescence and improve frailty in adult survivors of childhood cancer	NCT04733534	Frailty childhood cancer	Dasatinib + Quercetin, fisetin
Dasatinib plus quercetin for accelerated aging in mental disorders	NCT05838560	Schizophrenia aging, premature	Dasatinib + Quercetin
Senolytic therapy to modulate progression of Alzheimer's disease	NCT04063124	Alzheimer's disease	Dasatinib + Quercetin
Senolytic Therapy to Modulate the Progression of Alzheimer's Disease (SToMP‐AD) study	NCT04685590	Alzheimer's disease, early onset mild cognitive impairment	Dasatinib + Quercetin
Safety and feasibility of dasatinib and quercetin in adults at risk for Alzheimer's disease	NCT05422885	Alzheimer's disease	Dasatinib + Quercetin
Quercetin in coronary artery by‐pass surgery	NCT04907253	Coronary artery disease	Quercetin
Single nuclei RNA‐seq to map adipose cellular populations and senescent cells in older subjects	NCT05653258	Healthy lifestyle obesity	Dasatinib quercetin
Dasatinib and low intensity chemotherapy for Ph + acute lymphoblastic leukemia	NCT02888990	Leukemia, lymphoblastic, acute	Dasatinib
Adding ruxolitinib to a combination of dasatinib plus dexamethasone in remission induction therapy in newly diagnosed philadelphia chromosome‐positive acute lymphoblastic leukemia patients aged 40 years or older	NCT02494882	Acute lymphoblastic	Ruxolitinib, dasatinib dexamethasone
Senolytics to improve osteoporosis therapy	NCT06018467	Osteopenia, osteoporosis	Dasatinib quercetin, nicotinamide riboside
Quercetin's effect on bone health and inflammatory markers	NCT05371340	Osteoporosis, postmenopausal	Quercetin
Optimization of TKIs treatment and quality of life in Ph + CML patients ≥60 years in deep molecular response	NCT02326311	Leukemia chronic myeloid	Imatinib nilotinib dasatinib
Dasatinib plus anti‐CD19/CD22 bispecific CAR‐T cell therapy for elderly Ph‐positive ALL patients	NCT05523661	Ph Positive	Dasatinib plus anti‐CD19/CD22 CAR‐T cells
Study of dasatinib and all‐trans retinoic acid for relapsed/refractory and/or elderly patients with acute myelogenous leukemia (AML) or myelodysplastic syndrome	NCT00892190	Acute myelogenous leukemia myelodysplastic syndrome	Dasatinib (SPRYCEL) All trans retinoic acid (VESANOID)
Senescence in chronic kidney disease	NCT02848131	Chronic kidney disease	Dasatinib, quercetin
Anti‐inflammatory and antioxidant effects of resveratrol on healthy adults	NCT01492114	Chronic subclinical inflammation redox status	Resveratrol
Effects of resveratrol on inflammation in type 2 diabetic patients	NCT02244879	Type 2 diabetes mellitus inflammation insulin resistance	Resveratrol
Effect of resveratrol on age‐related insulin resistance and inflammation in humans	NCT01354977	Type 2 diabetes mellitus insulin resistance	Resveratrol
Resveratrol for improved performance in the elderly	NCT01126229	Memory	Resveratrol
Influence of caloric restriction and resveratrol in the sirtuin system in women and men aged 55–65 years	NCT01668836	Vascular system injuries lipid metabolism disorders endothelial dysfunction	Resveratrol caloric restriction
Impact of resveratrol on brain function and structure	NCT02621554	Healthy	Resveratrol
Resveratrol to enhance vitality and vigor in elders (REVIVE)	NCT02123121	Mitochondrial function physical function	Resveratrol 1000 mg/day resveratrol 1500 mg/day vegetable cellulose
Effects of dietary interventions on the brain in mild cognitive impairment (MCI)	NCT01219244	Mild cognitive impairment	Caloric restriction omega‐3 supplementation resveratrol supplementation

### 2.2 Quercetin

Quercetin (3,3′,4′,5,7‐pentahydroxyflavone) is composed of three benzene rings surrounded by five hydroxyl groups commonly found in various flowers, stems, and vegetables such as red onions and kale (Li et al., 2016). The name is derived from the Latin *quercetum*, meaning “oak forest.” Quercetin is partially soluble in water at high temperatures (Abraham & Acree, 2014). Less stable than fisetin, quercetin is more susceptible to degradation at alkaline pH and at high temperatures (Wang et al., 2016) (Table 1).

Quercetin is known for its potent antioxidant activity (Morand et al., 1998) and cancer‐prevention properties (Hashemzaei et al., 2017). Quercetin's activity involves multiple signaling pathways, including estrogen receptor signaling, mTOR, NF‐κB, PI3k/Akt, p53/p21/serpine, and HIF‐1α pathways (Reyes‐Farias & Carrasco‐Pozo, 2019). Quercetin can prolong the lifespan of HFL‐1 primary human fibroblasts and induce a rejuvenated phenotype in senescent HFL‐1 cells (Chondrogianni et al., 2010). Quercetin treatment decreased the percentage of β‐galactosidase positive cells in human fibroblasts (Chondrogianni et al., 2010). Moreover, quercetin's senolytic effect was demonstrated in vitro and in vivo (Zhu et al., 2015). Quercetin induced apoptosis in senescent human umbilical vein endothelial cells (HUVECs) and senescent mice bone marrow‐derived mesenchymal stem cells (BM‐MSCs), mainly via the PI3k/Akt and p53/p21/serpine pathways, but was less effective against senescent human preadipocytes (Zhu et al., 2015). Quercetin may also act as a senomorphic since it can slow the progression of intervertebral disc degeneration (IDD) through the Nrf2‐mediated suppression of NF‐κB activity (Shao et al., 2021).

The combination of quercetin and dasatinib—a second‐generation tyrosine kinase inhibitor originally approved for myeloid leukemia treatment in 2010—led to deactivation of survival‐promoting and anti‐apoptotic pathways of senescent cells (Zhu et al., 2017). In the clinics, dasatinib with quercetin was relatively safe and effective in mitigating physical dysfunction in patients with idiopathic pulmonary fibrosis (Justice et al., 2019). The combination of these two agents reduced inflammation by decreasing levels of circulating SASP factors. A decline in the abundance of senescent cells in the skin and adipose tissue of patients with diabetes‐related kidney diseases was reported (Hickson et al., 2019). Despite these positive outcomes, a significant adverse effect was reported in association with dasatinib and quercetin combined, associated with bacterial multifocal pneumonia and pulmonary edema (Justice et al., 2019). The implementation of this senolytic regimen in clinical practice has been limited due to the incomplete understanding of its systemic effects, uncertainties about the administration of quercetin, and potential interference with other biological pathways (Mbara et al., 2022). It is worth noting that several physicochemical features of quercetin, including chemical instability, low water solubility, and poor bioavailability, limit its use (Wang et al., 2016). Despite the numerous reports on quercetin's potential use as a senolytic or senomorphic agent, the mechanisms of this polyphenol remain unclear.

### 2.3 Resveratrol

Resveratrol (3,5,4′‐trihydroxy‐trans‐stilbene) is a naturally occurring polyphenol found in over 70 plants with wide‐ranging biological properties (Salehi et al., 2018). Its abundance in flora commonly increases in response to stress, UV radiation, fungal infection, etc. The largest significant dietary source of resveratrol is red wine (Kasiotis et al., 2013). Its structure consists of two benzene rings connected by an isopropylene moiety to produce a compact ring structure separated by a double bond (Table 1). It is a stilbenoid, a derivative of stilbene, available in both cis‐(Z) and trans‐(E) geometric isomers. When exposed to UV radiation, the trans‐form can photoisomerize into the cis‐form. The latter is more stable and exhibits greater bioactive properties (Bernard et al., 2007).

Resveratrol is well known for its antioxidant (Xia et al., 2017) and anticancer effects (Ko et al., 2017). Additionally, it exhibits anti‐neuroinflammatory properties, protecting against memory impairment in a rat cerebral palsy model (Calado et al., 2023). Resveratrol has been reported to significantly inhibit several SASP factors such as interleukin‐1β (IL‐1β), ‐8 (IL‐8), and tumor necrosis factor‐alpha (TNF‐α) through its inhibition of NF‐κB activation (Liu et al., 2018). Resveratrol can activate sirtuin 1 (SIRT1) (Figure 2), a class III histone deacetylase, which modulates DNA repair, metabolism, oxidative stress response, mitochondrial function, and biogenesis (Iside et al., 2020). Resveratrol treatment was found to reduce the expression of inflammatory factors NF‐κB, p65, receptor for advanced glycation end products (RAGE), and NADPH oxidase 4 (NOX4) in a mouse diabetic nephropathy model (Xian et al., 2020), suggesting its potential role in mitigating chronic inflammation. Additionally, multiple studies have found that resveratrol pretreatment mitigated ischemic injury by preventing neuronal loss and cognitive deficits (Arteaga et al., 2015; Della‐Morte et al., 2009; Xu et al., 2019; Yu et al., 2017). The anti‐inflammatory effect of resveratrol has been linked to a few signaling pathways such as the arachidonic acid pathway, NF‐κB, and MAPK (Figure 2) (Meng et al., 2021).

Resveratrol helps in preventing age‐related diseases by activating Nrf2 and boosting the activity of antioxidant enzymes such as superoxide dismutase (SOD) and catalase, thereby reducing the generation of ROS and subsequently decreasing oxidative stress (Zhou et al., 2021). Resveratrol can improve insulin sensitivity by decreasing ROS and activating the Akt pathway in Type 2 diabetes patients (Brasnyó et al., 2011). Several clinical trials investigating resveratrol in a variety of diseases and conditions are listed in Table 2. Resveratrol affects multiple cancer stages ranging from initiation to progression through a plethora of signal transduction pathways such as Nrf2 (Alavi et al., 2021) and SIRT1/p38/MAPK expression (Bian et al., 2022). While resveratrol can prevent senescence in healthy cells, resveratrol can also lead to beneficial cancer cell senescence (Bian et al., 2022; Ji et al., 2018), and has been shown to be cytotoxic against multiple human tumor cell lines (Ko et al., 2017).

### 2.4 Other polyphenols

Among other agents presented in Table 1, the following also merit a mention. Luteolin plays a significant role as an antioxidant, anti‐inflammatory, and anticancer agent, scavenging free radicals and suppressing neuroinflammation (Mbara et al., 2022; Shi et al., 2004). Berberine, a natural alkaloid commonly used to treat diarrhea, has also been shown to rescue senescent cells, extend health span and improve fur density and behavioral activity in chemotherapy‐treated and aged mice (Dang et al., 2020). Curcumin, a phytochemical found in turmeric, has been suggested to extend lifespan in animals (Shen et al., 2013), but it can induce senescence itself in vascular smooth muscle cells (Grabowska et al., 2019) and fibroblasts (Chu et al., 2023; Grabowska et al., 2019).

Curcumin decreased the expression of pro‐inflammatory cytokines by reducing binding of AP‐1, NF‐IL6, ETS, and NF‐κB to IL‐1α, IL‐1β, TNF‐α, and IL‐6 promoters, respectively (Das & Vinayak, 2018). Curcumin inhibits superoxide anion‐induced pain‐like behaviors in mice, as well as leukocyte migration, by increasing Nrf2 and decreasing NF‐κB activation. This modulation was associated with lower levels of IL‐1β and TNF‐α as well as increased levels of IL‐10 (Fattori et al., 2015). Curcumin's low toxicity is one of its key advantages; however, it has poor oral bioavailability, low solubility in water, and fast breakdown rates, making its administration challenging (Purpura et al., 2018).

Taken together, polyphenols modulate multiple signaling pathways depending on cell types and cellular context. It is yet to be shown if these polyphenols could be used as therapeutic interventions in pathologies implicating senescence.

## 3 SENOTHERAPEUTICS IN CLINICAL TRIALS

Early pilot trials showed that senolytics were helpful in eliminating senescent cells, reducing frailty, and decreasing inflammation in humans (Kirkland & Tchkonia, 2020). As a result, only a few senolytics have been tested in human clinical trials. Senomorphics, on the other hand, have yet to be tested clinically. In contrast to senolytics, senomorphics must be taken on a regular basis for an extended period of time to display their full benefits because they intervene in important pathways and have potentially more serious side effects (Song et al., 2020). Table 2 summarizes the senolytic drugs used in clinical studies.

The initial human trial involving co‐administration of dasatinib and quercetin was conducted on patients with idiopathic pulmonary fibrosis (IPF) (Justice et al., 2019). Posttreatment assessment included tests of physical function, encompassing walking endurance, gait speed, and chair rise, all of which showed significant improvement. Additionally, alterations in the expression of various SASP factors, such as matrix proteases, miRNAs, and pro‐inflammatory cytokines, were noted (Justice et al., 2019). Furthermore, the co‐administration of dasatinib and quercetin was assessed in diabetic patients with chronic kidney disease, revealing optimal reduction in senescent cell burden in vivo (Hickson et al., 2019). Specifically, adipose tissue biopsies conducted before and after treatment demonstrated a decrease in the number of p16‐positive senescent cells. Additionally, tissue inflammation, as indicated by reduced macrophage infiltration, and several components of the SASP such as IL‐1α, IL‐6, and MMPs, among others, were diminished in patients' plasma (Hickson et al., 2019). There is compelling evidence suggesting that this combined treatment may effectively address age‐related frailty, obesity, osteoporosis, and insulin resistance.

## 4 TOXICOLOGY AND LIMITATIONS OF POLYPHENOL THERAPY

The aim of many studies on polyphenols was to investigate the protective effects of these compounds against diseases or harmful drugs. However, there remains a lack of comprehensive research on the potential toxicity of these dietary components. Flavonoids, for instance, may disturb various stages involved in the progression of malignant tumors, including protecting DNA from oxidative stress, triggering carcinogen‐detoxifying mechanisms, and inhibiting carcinogen activation (Birt et al., 2001; Galati et al., 2000; Ren et al., 2003). Dietary polyphenols can act as pro‐oxidants in systems containing redox‐active metals. When exposed to oxygen, transition metals like copper (Cu) and iron (Fe) facilitate the redox cycling of phenolics, resulting in the generation of ROS and phenoxyl radicals. These can damage DNA, lipids, and other essential biological molecules (Decker, 1997; Li & Trush, 1994; Yamanaka et al., 1997). The most ubiquitous dietary flavonoid, quercetin, consists of a catechol B ring that can be oxidized by tyrosinase, hydrogen peroxide, and peroxidases. This process leads to the formation of quinone/quinone methide intermediates, which subsequently react with glutathione (GSH), resulting in quercetin glutathionyl adducts (Awad et al., 2000, 2002; Galati et al., 2001). Studies have shown that quercetin can covalently interact with cellular DNA and proteins in human intestinal Caco‐2 cells and hepatic Hep G2 cells (Walle et al., 2003). Although quercetin is an antioxidant, several researchers have shown the opposite. Specifically, certain flavonoids are metabolized by ROS into compounds that covalently interact with essential target macromolecules.

Extensive research has been conducted on the inhibition of cytochrome P450s (CYPs) by flavonoids because of their potential as agents that block the initiation phase of carcinogenesis (Doostdar et al., 2000). However, this property may pose a risk in the context of flavonoid‐drug interactions. Flavonoids with hydroxyl groups hinder CYP activity, whereas those without may activate the enzyme (Hodek et al., 2002). Depending on their structures, concentrations, or experimental conditions, flavonoids can both inhibit and stimulate human CYPs. For instance, α‐Naphthoflavone inhibits human CYP1A1 and 1A2 (Tassaneeyakul et al., 1993) but stimulates CYP3A4 (Guengerich et al., 1994). Understanding the interplay between flavonoids and CYP3A4, the primary human hepatic and intestinal CYP responsible for metabolizing 50% of drugs, is crucial (Hodek et al., 2002).

Fisetin undergoes phase II metabolism through glucuronidation and methylation (Touil et al., 2011). Catechol‐O‐methyltransferase (COMT) converts fisetin to geraldol via O‐methylation, while UDP‐glucuronosyl transferase (UGT) forms a glucuronide conjugate. This indicates that fisetin metabolism is not mediated by cytochrome P450 (CYP), despite its ability to inhibit cytochrome P450 in human liver microsomes (Shrestha et al., 2018). Fisetin acts as a competitive inhibitor against CYP2C9, with a ki value of less than 2.2 μM, implying its binding to the substrate‐binding site. Additionally, it exhibits a strong inhibitory effect on CYP2C19 and CYP1A2, and a weaker inhibition on CYP3A4 and CYP2D6 (Jung & Lee, 2014; Si et al., 2009). This can result in drug interactions when co‐administering flavonoids with other drugs, potentially causing increased toxicity and reduced therapeutic effect (Tang & Stearns, 2001). The inhibition of CYPs by flavonoids could impede the metabolism and elimination of drugs, increase their plasma accumulation in vivo and potential toxicity. Additionally, specific CYPs can be induced by flavonoids, leading to the metabolic activation of carcinogens. For instance, 2,3,7,8‐tetrachlorodibenzo‐p‐dioxin induces CYPs by binding to aryl hydrocarbon receptor (AhR) (Kohn et al., 2001). As a result, the activities of the CYP1 family enzymes, responsible for the activation of carcinogens like benzo[a]pyrene, aflatoxin B1, and 7,12‐dimethylbenz[a]anthracene, are elevated (Omiecinski et al., 1999). Several flavonoids, such as galangin, quercetin, diosmin, and diosmetin, have been identified as AhR agonists, inducing the activities of CYP1A1 and CYP1A2 (Waller & McKinney, 1995). CYP1A2 is involved in the metabolic activation of aromatic amine carcinogens, while CYP1A1 is associated with the activation of polycyclic aromatic hydrocarbon carcinogens. The binding affinities of flavonoids to AhR are primarily dependent on their structure, favoring planar aromatic compounds with minimal bulky substituent groups. Similarly, some flavonoids like flavone, tangeretin, and synthetic h‐naphthoflavone act as inducers of CYP1A1/2 and to some extent CYP2B1/2 (Hodek et al., 2002). Flavanones, on the other hand, serve as specific inducers of CYP2B1/2 (Canivenc‐Lavier et al., 1996).

## 5 NANOCARRIERS FOR SENOTHERAPEUTICS

### 5.1 Nanocarriers to overcome limitations of polyphenol therapy

A plethora of formulations has been employed to improve the bioavailability and relatively poor pharmacokinetics of “free‐form” phytochemicals (Sa et al., 2023). The lack of target selectivity is often described as a hurdle or setback in the classic “one drug, one target” approach to drug discovery, and indeed, there have been increasing calls to investigate drugs that have multiple targets (Casas et al., 2019; Talevi, 2015), which would be beneficial in pathologies involving a multiplicity of pathways such as senescence. In fact, the pleiotropic neuroprotective effects of senolytics such as resveratrol may provide increased benefits against cellular senescence and senescence‐associated hallmarks such as cognitive decline or neurodegenerative diseases (Griñán‐Ferré et al., 2021). Phytochemicals have been investigated in concert with classic anticancer agents to enhance their therapeutic index and prevent anticancer drug resistance (Li et al., 2022; More et al., 2021; Zhao et al., 2016). However, fast absorption, isomerization, autooxidation, poor water solubility, and presystemic metabolism have restricted the use of many senolytic drugs in vivo in their free form.

**FIGURE 3:**
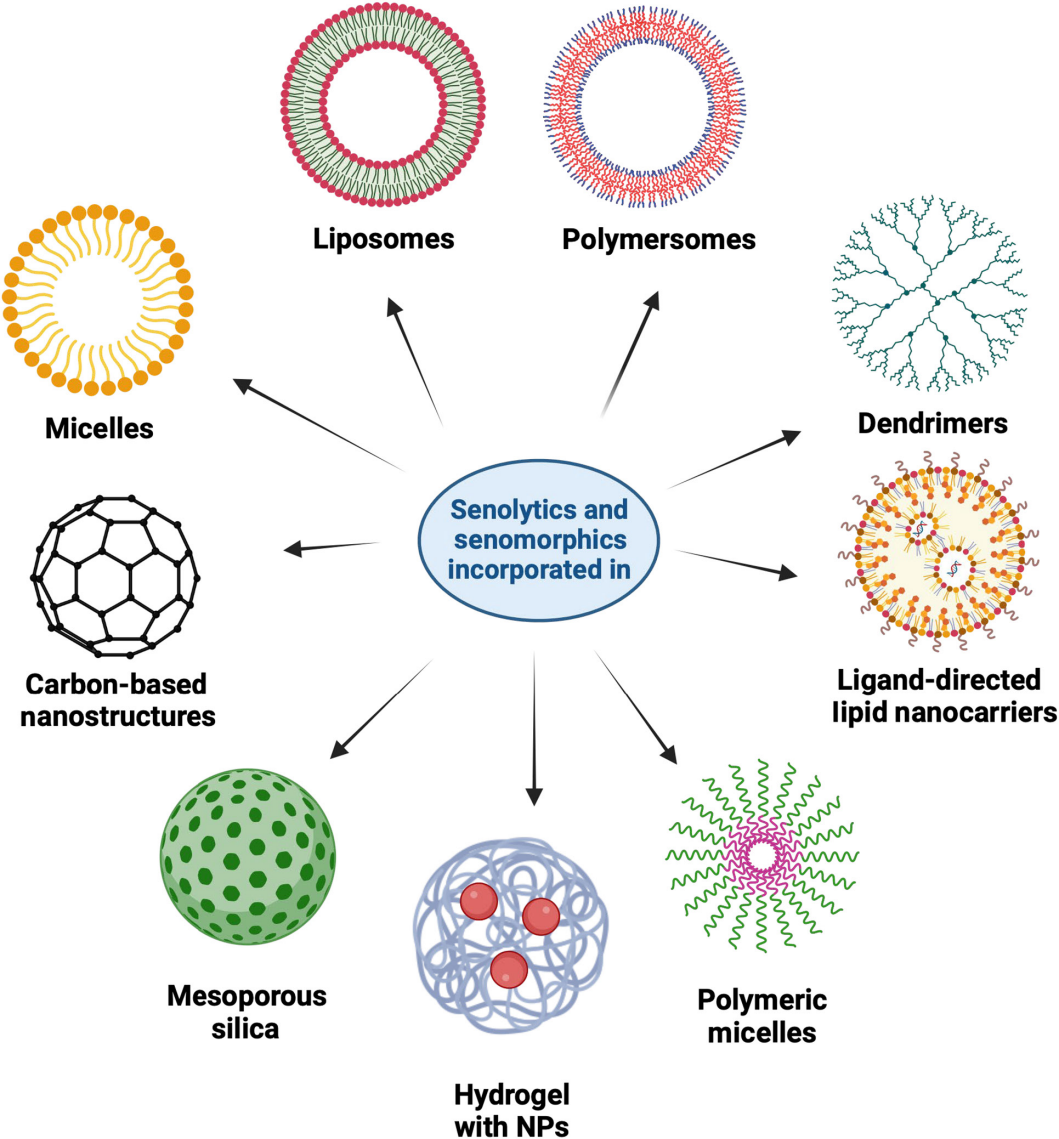
Nanocarriers developed for the delivery of senolytics and senomorphics. Several nanocarriers have been developed for the delivery of senolytic and senomorphic drugs such as resveratrol, fisetin, and quercetin (Table 3 presents a summary of investigated nanoformulations). Created using BioRender.

Flavonoids, such as fisetin, quercetin, and resveratrol, could offer considerable health benefits, and their senotherapeutic properties are highlighted in this review. However, these polyphenols generally suffer from very poor pharmacokinetics, and nanocarriers provide an advantageous platform to address these challenges and maximize their therapeutic efficacy. A variety of nanoformulations have been developed for this purpose, and an overview of these is provided in Figure 3. We include below a brief description of encapsulating polyphenols into some of these carriers.

*Liposomes*: It has recently been suggested that the ability of polyphenols to incorporate into lipid membranes is influenced by their chemical structure (Uekusa et al., 2008), and there is a strong correlation between the partition coefficient across the membrane and the integration of polyphenol into liposomes. For example, Patra et al. reported an interaction involving –OH groups of the bile salt (sodium cholate) and curcumin incorporated into dipalmitoyl phosphatidylcholine (DPPC) liposomes (Patra et al., 2013). Polyphenols could interact with bilayers in liposomes to enhance their loading efficiency, as well as influence the release rate. These interactions could lead to (i) the hydrophobic bilayer of the membrane separating the less polar molecules and (ii) the more hydrophilic polyphenols forming hydrogen bonds with the polar head groups of lipids and the membrane interface (Oteiza et al., 2005). This phenomenon is similar to cholesterol, a common component of the lipid bilayer, which has its hydroxyl groups available for hydrogen bonding with the aqueous environment.
*Polymeric nanoparticles*: Self‐assembled structures from amphiphilic polymers in an aqueous medium (micelles) are composed of a hydrophobic core and hydrophilic shell. In addition to simple physical entrapment of senolytics into the core, polyphenols could utilize noncovalent interactions (hydrogen bonds and hydrophobic interactions) with the core polymeric architectures and enhance the loading efficacy of such carriers. Polymersomes are self‐assembled vesicular structures that can stabilize both hydrophobic and hydrophilic drugs and are more stable than liposomes (Zhang & Zhang, 2017).
*Dendrimers*: Dendrimers are nanometer‐sized, hyperbranched, spherical synthetic macromolecules and possess several terminal groups like –NH_2_ that can serve as attachment sites for drug molecules, enabling them to efficiently transport drugs. Moreover, polysulfated dendrimers have shown therapeutic potential, notably through anti‐inflammatory effects (Maysinger et al., 2023). Dendrimers possess several binding sites for hydrophobic, hydrophilic, cationic, anionic drugs and have nanoscale dimensions, dispersity, stability, and chemical surface modification capabilities due to their unique architecture (Svenson & Tomalia, 2012; Tomalia et al., 2012).
*Hydrogel*s: Hydrogels contain a three‐dimensional cross‐linked polymeric network that can swell in an aqueous medium and biological fluids. The constituent polymeric components can be customized to meet desired needs, which include active or passive targeting, loading capacity, and controlled release of bioactive components (Micale et al., 2020). A recent paper discussed details related to hydrogel derived from poly‐vinylpyrrolidone (PVP) and Fe^3+^, and loading it with varied polyphenols or cross‐linking these by hydrogen bonding and coordination interactions (Hu et al., 2022).
*Mesoporous silica*: Mesoporous silica nanoparticles (MSNs) have been extensively studied as carriers because of their biocompatibility, stability, pore size, and surface functionalization, which allow high polyphenol loading (Chircov et al., 2020; Huang et al., 2014). A variety of polyphenols can be loaded into mesoporous silica, which was ascertained using a variety of techniques, including BET, FT‐IR, and thermogravimetric analyses (Petrisor et al., 2022).

An analysis of the varied parameters of nanocarriers that have been utilized in encapsulating senotherapeutics is provided in Table 3.

**TABLE 3:** Overview of nanocarriers used to encapsulate senolytics.

Nanocarriers	Structure	Properties	Average size	Pros and cons
Polymeric micelles	Core‐shell structure formed by amphiphilic polymers	The hydrophilic shell stabilizes micelles and prevents aggregation, while the hydrophobic core encapsulates hydrophobic drugs	10–100 nm (Bagheri et al., 2018)	Good aqueous solubility, controlled release, good therapeutic action, but desires synthetic articulation
Dendrimers	Monodisperse, highly branched macromolecules consisting of an outer functional group, inner layers, and core	Polyvalency, self‐assembled, electrostatic interactions, structural diversity and specificity	1–15 nm. (Mittal et al., 2021)	Low cytotoxicity, chemical stability, solubility, protection of drug but suffers from toxicity and biodistribution
Liposomes	Liposomes are spherical lipid bilayer vesicles resembling cell membranes	Spherical lipid vesicles, biodegradable, biologically inert, and nonimmunogenic lipid	50–500 nm (Nsairat et al., 2022)	High therapeutic efficiency, flexible, nontoxic but suffers from short half‐life and poor solubility
Hydrogels	Hydrogels, made of hydrophilic polymers with cross‐links, absorb water up to hundreds of times their own weight	Controlled degradability, cross‐linking gives good aqueous solubility	5–100 nm (Li & Mooney, 2016)	Biocompatible, potential, hydrophilicity but suffers from nonspecific drug release in diffusion‐controlled mechanism
Mesoporous silica NP	Inorganic nanoparticles with porous structure and large surface area	Tunable particle size, uniform pore size	30–300 nm (Wu et al., 2013)	High drug loading capacity, thermally stable, biocompatible but interaction between the surface density of silanol groups and the phospholipids on the surface of red blood cell membranes leads to hemolysis

A typical nanocarrier utilized for the purpose has its own advantages and disadvantages in addressing key issues related to polyphenol pharmacokinetics and improving its therapeutic potential. These are elaborated in Table 4 and considering the variability of data available at this stage, a comparative analysis of these is cumbersome. The choice of a particular nanocarrier is guided by several key factors, including an understanding of senescence, the desired senolytic or senomorphic to be physically encapsulated, and its controlled release.

**TABLE 4:** Senolytic agents in nanocarriers.

Natural compound	Type of nanocarrier	Size (nm)	Condition	Biological effect	Payload release	References
Fisetin	Nanostructured lipid nanocarriers (NLCs)	135.0 ± 5.5	Advanced and metastatic melanoma	Enhanced expression of Nrf2/NQO1 genes; upregulation of BAX mRNA expression	The formulation stable over period of 60 days and demonstrated sustained release of drug over 96 h	(Kumar et al., 2023)
	Monomethyl poly‐(ethylene glycol) − poly(ε‐caprolactone) (MPEG−PCL) copolymer micelle	22.4 ± 3.0	Colon cancer	Enhanced cytotoxicity, cellular uptake, and apoptosis in CT26 cells; superior tumor growth suppression compared to free fisetin	Sustained and prolonged in vitro release behavior	(Chen et al., 2015)
	Pluronic F127‐Folic acid conjugated micelles	103.2 ± 6.1	Breast cancer	Significant inhibition of folate overexpression; reduced tissue toxicity in liver, kidney, heart, and spleen	80% fisetin released at pH 5.3 over 5 days; 50% at pH 7.4	(Pawar et al., 2018)
	Poly‐(lactic acid) (PLA) NPs	226.85 ± 4.78	Breast cancer	2‐fold decrease in tumor volume; 1.6‐fold decrease in tumor weight	Slow and controlled release; 80% drug release over 4 days	(Feng et al., 2019)
	Chitosan(CS)‐based NPs	363.1 ± 17.2	Osteoarthritis	Inhibition of inflammatory responses (IL‐6 and TNF‐α); reduction in tissue toxicity	8% fisetin detected within 48 h in vitro	(Nabizadeh et al., 2023)
Quercetin	Fe_3_O_4_ NPs	140	Oxidative stress‐induced senescence	Promoted AMPK activity; decreased number of stress‐induced senescent cells and suppression of SASP (decreased IL‐8 and IFN‐β)	Not specified	(Lewinska et al., 2020)
	Aminopropyl functionalized mesoporous silica NPs (NH2‐MSN)	250 ± 50	Melanoma	Around 50% inhibition of cell proliferation at 72 h	Prolonged release and enhanced bioavailability of the included active molecule	(Sapino et al., 2015)
	Poly‐(lactic acid) (PLA) NPs	32 ± 8 to 152 ± 9	Breast cancer	Around 40% decrease in cell viability after 5 days	Controlled and sustained drug release	(Pandey et al., 2015)
	Nanostructured lipid carriers	32	Breast cancer	About 14% reduction in viability of MCF‐7 cells and MDA‐MB‐231 cells at 48 h	Sustained drug release	(Sun et al., 2014)
	Nanomicelles made from the copolymer, PEG‐derivatized phosphatidylethanolamine (PE)	15.4–18.5	Lung cancer	Increased tumor growth inhibition than free quercetin	Quercetin nanomicelles were stable at pH 1.2 and pH 7, with ∼30% release of quercetin	(Chiu et al., 2012)
Resveratrol	Nanostructured lipid carriers (NLCs)	103.48 ± 3.65 to 708.3 ± 12.48	Breast cancer	Increased permeation of resveratrol into the skin, improved bioavailability; enhanced anticancer activity in MDA‐MB‐231 breast cancer cell lines	Initial burst release observed, followed by sustained release	(Gadag et al., 2021)
	Gold NPs	40	Pancreatic ductal adenocarcinoma (PDAC)	Enhanced caspase‐mediated apoptosis in pancreatic PANC‐1 cells through the intrinsic mitochondrial apoptotic pathway	Rapid release within 96 h at pH 5.0, suggesting efficient delivery to the tumor through blood vessels	(Lee et al., 2022)
	Mesoporous silica NPs	69.1 ± 5.2	Melanoma	Decrease in cell viability with increasing concentrations of resveratrol‐loaded MSNs against different human melanoma cell lines	Increased in vitro release in an acid medium (pH 5.2)	(Marinheiro et al., 2021)
	Fusogenic liposomes	–	Cerebromicrovascular endothelial cells isolated from aged rats	Enhanced delivery of resveratrol into aged cells and rapid activation of Nrf2‐driven antioxidant defense mechanisms	Efficient release of resveratrol when fused to cell membranes	(Csiszár et al., 2015)
Curcumin + Resveratrol	Dendritic polymer NPs	<200	Neuroblastoma	Synergism induces increased mitochondrial disruption, affecting intracellular calcium release, and causing cancer cell death	Slow‐release kinetics over time	(Ben‐Zichri et al., 2022)
Dasatinib	NanoMIPs molecularly imprinted NPs	70–200	Bladder cancer	Decreased number of senescent cancer cells	Not specified	(Ekpenyong‐Akiba et al., 2019)
Dasatinib + Quercetin	WAT‐targeted liposomes	145	Senescence and lipolysis	Eliminated senescent cells and reduced lipolysis	Over 80% of D and nearly all of Q were steadily released from liposomes over 50 hours without any sudden bursts	(Tang et al., 2024)

These drug delivery systems can allow for the targeted transport and delivery of therapeutic agents while providing protection from early degradation. The use of nano‐based drug delivery systems has allowed for increased efficacy of naturally occurring bioactive molecules such as quercetin (Attar et al., 2023), curcumin (Rahimi et al., 2016), resveratrol (Chung et al., 2020), and berberine (Javed Iqbal et al., 2021), among many others (Adamczyk‐Grochala & Lewinska, 2020). Nanostructures including polymers, peptides, or surfactants may improve biocompatibility while limiting possible immunogenicity. Coating nanostructures with polyethylene glycol (PEG), termed PEGylation, is a widely used modification to allow for increased bioavailability and improved drug delivery (Suk et al., 2016). While PEG is a versatile polymer with favorable properties and widespread use in preclinical and clinical studies, some have erroneously claimed that PEG is nonimmunogenic. PEG has been found to cause slight immunogenicity when introduced as components of nanocarriers, though such reactions were rare (Ibrahim et al., 2022; Kozma et al., 2020). Overconsumption of PEGs as laxatives led to an immune response in a subpopulation of patients. Still, the benefits overweigh the limitations, and identifying individuals' immunoresponsiveness to PEG is feasible and should be implemented.

Existing recommendations for the identification of senescent cells present several challenges (Gil, 2023), some of which could be overcome by nanotools. Senescence‐associated β‐galactosidase often results in nonspecific staining and limits in vivo identification of senescent cells. Cargo coated with galacto‐oligosaccharides was found to be preferentially released within senescent cells while reducing cardiotoxic side effects of doxorubicin (Adamczyk‐Grochala & Lewinska, 2020; Muñoz‐Espín et al., 2018).

### 5.2 Examples of senotherapeutics in nanocarriers

An overview of the most common nanocarriers and the polyphenols they incorporate, along with their biological effects, is summarized in Table 4.

Polyphenols are typically delivered using micelles and liposomes, although there are examples of other nanocarriers. While micelles and liposomes are attractive drug delivery systems, they come with certain limitations. First, due to the necessity of administering large amounts of polyphenols to achieve a therapeutic dose, a high quantity of polymer is required to maintain its concentration above the critical micelle concentration (CMC). Second, controlling the rate and site of polyphenol release in the complex biological system is challenging. Despite these limitations, both micelles and liposomes have demonstrated enhanced bioavailability of polyphenols.

Lipid‐based nanocarriers, such as liposomes and micelles (Figure 3), allow for increased bioavailability of senotherapeutics. In rats, oral administration of a curcumin‐phospholipid complex maintains the effective concentration of curcumin for longer periods of time compared to free curcumin (Maiti et al., 2007). In one study, resveratrol was encapsulated in “fusogenic” liposomes, vesicles capable of fusing with cell membranes, and used to treat cerebromicrovascular endothelial cells isolated from aged rats (Csiszár et al., 2015). Nanoliposomes have also been studied as a transdermal drug delivery system functionalized with resveratrol to delay aging in a dermatological 3D skin model (Zhang, Chen, et al., 2023). Nanocarriers can also be fabricated from inorganic substrates. In vitro treatment with Fe_3_O_4_ nanoparticles functionalized on its surface with quercetin on oxidative stress‐induced senescence in human fibroblasts, promoted AMPK activity and nonapoptotic cell death, while decreasing the number of senescent cells and the level of secreted IL‐8 and IFN‐β (Lewinska et al., 2020).

Ekpenyong‐Akiba et al., 2019 introduced molecularly imprinted nanoparticles (nanoMIPs) as a novel technology for targeting senescent cells. In this method, the template molecule (B2M) is covalently immobilized on a solid support, initiating polymerization to form polymer nanoparticles. In vitro experiments in this study show that B2M‐targeted nanoMIPs can selectively recognize senescent cells without causing toxicity. Fluorescently labeled nanoMIPs preferentially accumulated in senescent cells, indicating their potential for diagnostic and therapeutic applications. In vivo studies revealed that B2M‐targeted nanoMIPs could detect senescent cells in aged mice. Moreover, the accumulation of nanoMIPs in the abdominal cavity of old mice suggested a higher presence of senescent cells in the gastrointestinal tract, liver, and spleen.

Tang et al. (2024) reported that senescent white adipose tissue (WAT) could promote age‐related hepatic steatosis by increasing lipolysis of WAT and the influx of free fatty acids (FFA) into hepatocytes. WAT‐targeted liposomes were developed to deliver dasatinib + quercetin specifically to adipose tissue. These liposomes efficiently eliminated senescent cells in WAT without significant cytotoxicity to normal cells. Moreover, WAT‐targeted liposomes decreased lipolysis of WAT during aging and reduced circulating FFA levels. In vivo studies using naturally aged mice showed that WAT‐targeted liposomes were more effective in eliminating senescent cells in WAT, reducing lipolysis, and decreasing hepatic lipid accumulation compared to conventional administration of dasatinib + quercetin.

## 6 CONCLUSION AND FUTURE PERSPECTIVES

This mini‐review highlights properties, limitations, and applications of selected senotherapeutic polyphenols with and without nanodelivery vehicles. Studies on polyphenols as senolytics and senomorphics are only beginning to make their way to preclinical and clinical trials. Due to the limited solubility of polyphenols in aqueous media, several nanodelivery systems have been fabricated to make polyphenols more suitable for systemic administration and/or prevent their premature degradation, particularly when intended for oral administration. Although these are valuable contributions to improving applications of senolytics and senomorphics, it will require many more investigations to take full advantage of polyphenols in the context of senescence and “senopathies.”

We have summarized some preclinical and clinical studies with polyphenol senotherapeutics. Most cytotoxic and cytostatic anticancer agents cause senescence. Several key signaling pathways implicated in senotherapeutic effects are presented and briefly commented on. Recent studies have shown that in certain conditions and contexts, malignant, and nonmalignant cells with chronic senescence can acquire tumorigenic properties (Reimann et al., 2024; Schmitt et al., 2022). Thus, interventions which would prevent the switch toward tumor‐promoting conditions warrant further investigations. Pharmacological suppression or modulation of SASP might be beneficial to a certain extent in some patients, but it is unlikely that it would profoundly change tumor fate. A premature cancer cell senescence exerts an acute beneficial outcome, but chronic senescence is detrimental. Demarid's team proposes that senolysis seems to be the preferred therapeutic strategy with a higher probability of tumor eradication (Reimann et al., 2024; Schmitt et al., 2022).

However, a series of reliable senescence and aging biomarkers is needed for the evaluation of the effectiveness of senolytics. Pleiotropic effects of polyphenols as senolytic or senomorphic agents might be beneficial in some individuals but cannot be considered a universal pharmacological approach. More data are needed to show a causal relationship between the functional impairments of individual cells, tissues, organisms, and the effectiveness of senolytic or senomorphic agents. Senescent cells that resume proliferation are fundamentally different from those that never entered senescence. A dynamic progression to a postsenescent state comes with distinct functional and clinically relevant ramifications (Reimann et al., 2024). Artificial intelligence (AI) data combined with imaging and omics‐based analysis could be useful in predicting favorable intervention outcomes at a personalized level. It is a promising direction in better controlling senescence and slowing down aging, but we are not there yet!

## AUTHOR CONTRIBUTIONS

N.J., P.B., and A.S. conducted a literature review and collaboratively prepared the initial draft of the manuscript. N.J. and P.B. prepared the figures. N.J. and A.S. prepared the tables. N.J., P.B., A.S., A.K., and D.M. contributed to the editing of the manuscript. All co‐authors have read and agreed to the published version of the manuscript.

## FUNDING INFORMATION

Funding from the Natural Sciences and Engineering Research Council of Canada (NSERC; DM, RGPIN‐2020‐07011; AK, RGPIN‐2023‐03565) and the Fonds de Recherche du Québec – Santé (EuroNanoMed III – PLATMED) (FRQ‐S 294233).

## CONFLICT OF INTEREST STATEMENT

The authors have no conflict of interests to declare.

## Data Availability

This is a review article and data was referred to as reported in the cited articles. No separate data analysis was performed.
